# Case Report: A Novel Variant c.2262+3A>T of the *SCN5A* Gene Results in Intron Retention Associated With Incessant Ventricular Tachycardias

**DOI:** 10.3389/fmed.2021.659119

**Published:** 2021-08-04

**Authors:** Jie Yin, Jia Zhou, Jinlong Chen, Ting Xu, Zhongman Zhang, Han Zhang, Chang Yuan, Xueying Cheng, Yuming Qin, Bixia Zheng, Chunli Wang, Shiwei Yang, Zhanjun Jia

**Affiliations:** ^1^Department of Cardiology, Children's Hospital of Nanjing Medical University, Nanjing, China; ^2^Nanjing Key Laboratory of Pediatrics, Children's Hospital of Nanjing Medical University, Nanjing, China

**Keywords:** inherited arrhythmia, cardiac sodium channel, *SCN5A*, new donor site, abnormal RNA splicing

## Abstract

**Objective:** Voltage-gated sodium channel Na_v_1.5 encoded by the *SCN5A* gene plays crucial roles in cardiac electrophysiology. Previous genetic studies have shown that mutations in *SCN5A* are associated with multiple inherited cardiac arrhythmias. Here, we investigated the molecular defect in a Chinese boy with clinical manifestations of arrhythmias.

**Methods:** Gene variations were screened using whole-exome sequencing and validated by direct Sanger sequencing. A minigene assay and reverse transcription PCR (RT-PCR) were performed to confirm the effects of splice variants *in vitro*. Western blot analysis was carried out to determine whether the c.2262+3A>T variant produced a truncated protein.

**Results:** By genetic analysis, we identified a novel splice variant c.2262+3A>T in *SCN5A* gene in a Chinese boy with incessant ventricular tachycardias (VT). This variant was predicted to activate a new cryptic splice donor site and was identified by *in silico* analysis. The variant retained 79 bp at the 5′ end of intron 14 in the mature mRNA. Furthermore, the mutant transcript that created a premature stop codon at 818 amino acids [p.(R818^*^)] could be produced as a truncated protein.

**Conclusion:** We verified the pathogenic effect of splicing variant c.2262+3A>T, which disturbed the normal mRNA splicing and caused a truncated protein, suggesting that splice variants play an important role in the molecular basis of early onset incessant ventricular tachycardias, and careful molecular profiling of these patients will be essential for future effective personalized treatment options.

## Introduction

The inherited cardiac arrhythmias mainly including long QT syndrome (LQTS), short QT syndrome (SQT), catecholaminergic polymorphic ventricular tachycardia (CPVT), Brugada syndrome (BrS), and some other arrhythmias are often the underlying cause of sudden cardiac death (SCD) in young individuals (1, 2). Studies investigating SCD in young individuals (of <35 years of age) have estimated an incidence of 1.3–3.2 per 100,000 person-years, and there is a higher prevalence of SCD in males than in females (3–5). Although these inherited cardiac arrhythmias are closely associated with SCD, systematic population-based studies are lacking, and the real prevalence is largely unknown. These rare diseases, also called cardiac channelopathies, are results from mutations in several genes encoding ion channels or proteins involved in their regulation (6). Genetic variants in the sodium ion channel (such as *SCN5A*), calcium ion channel (such as *CACNA1C*), and potassium ion channel (such as *KCNQ1, KCNH2*) are more critical for inherited cardiac arrhythmias (6). The genetic defects lead to alterations in the morphology and duration of the cardiac action potential, and resulting syncope or a life-threatening arrhythmic episode in patients with such disorders. Management relies on pharmacological therapy, mostly β-adrenergic (such as metoprolol and propranolol) and sodium and transient outward current blockers (such as quinidine) (6, 7), or surgical interventions, including left cardiac sympathetic denervation (8). In the case of cardiac arrest or recurrent syncope, an implantable cardioverter defibrillator (ICD) is appropriate (9).

Cardiac voltage-gated sodium channels are transmembrane proteins located in the sarcolemma of myocytes that play critical roles in the normal electrical activity of the heart. The major pore-forming α-subunit (Na_v_1.5) of the cardiac sodium channel is encoded by the *SCN5A* gene. Genetic variants of *SCN5A* are involved in a wide variety of inherited arrhythmias including LQTS, SQTS, BrS, CPVT, and cardiac conduction system dysfunction (10, 11). Meanwhile, *SCN5A* variants are also found to be correlated with dilated cardiomyopathy (12, 13) and heart failure (14). The clinical courses of these arrhythmogenic disorders are diverse, ranging from asymptomatic to episodic syncope and even sudden cardiac death (SCD) in patients with normal cardiac structures. In recent years, genetic testing has been increasingly applied in clinical practice and is currently recommended for most inherited arrhythmia disorders. However, genetic screening of cardiac ion-channel genetic mutations may give negative results in many patients with positive electrocardiogram (ECG) phenotypes suggestive of cardiac channelopathies (15). Although electrophysiological and molecular genetics studies have provided insights into dysfunction and dysregulation of the cardiac sodium channel, including the identification of *SCN5A* variants in patients with inherited arrhythmia disorders over the last two decades, the genotype–phenotype relationships of cardiac sodium channelopathies remain unclear. A recent study of 442 patients with *SCN5A* variants found that 67.9% were asymptomatic at diagnosis, and 44.3% had negative ECG phenotypes (16). Many important issues require additional study to improve our understanding of the mechanisms underlying cardiac sodium channelopathies.

In this report, we reported a male infant patient with a history of recurrent VT, for whom whole-exome sequencing (WES) was used in early diagnoses of cardiac sodium channelopathies caused by *SCN5A* gene variants. A novel splice variant c.2262+3A>T was identified and predicted to be deleterious in mRNA splicing. Our minigene assay and Western blot verified the pathogenic effect of the novel splicing variant c.2262+3A>T, which retained partial intron 14 in mRNA and caused a truncated protein, suggesting that splice variant plays an important role in the molecular basis of early onset incessant ventricular tachycardias.

## Materials and Methods

### Case Description

**Figure 1:**
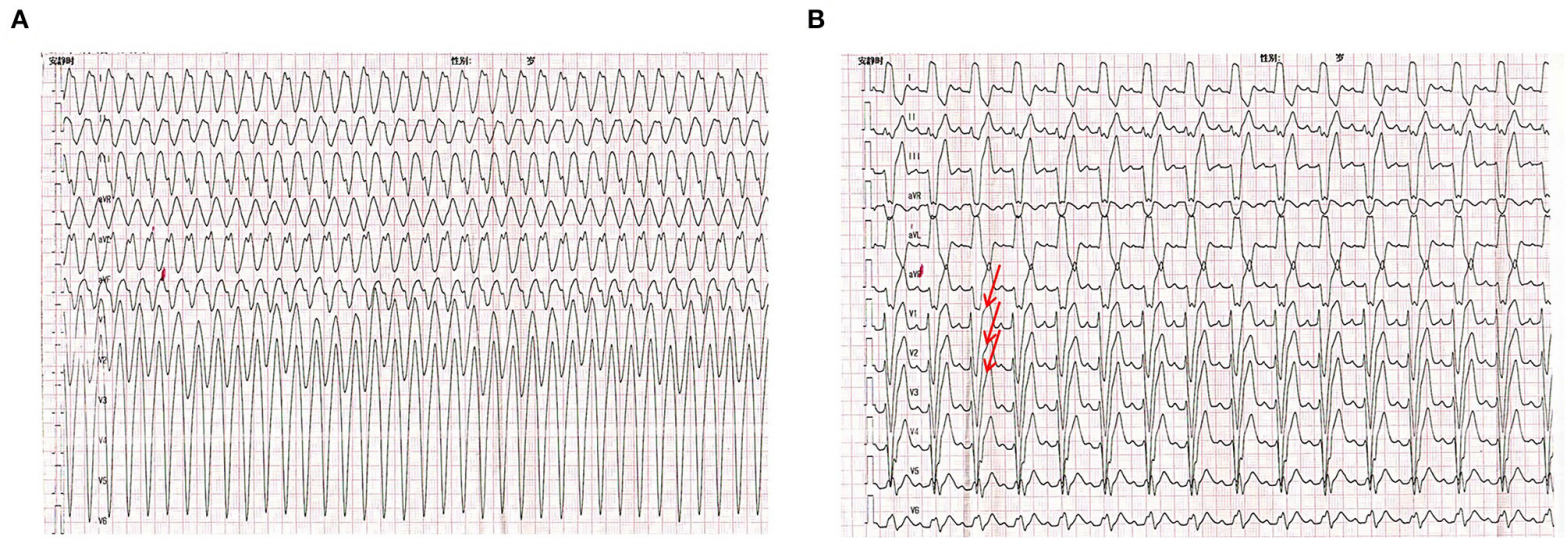
Electrocardiogram (ECG) of the proband. **(A)** The ECG of the proband showed ventricular tachycardia onset at a cycle length of 280ms. **(B)** Baseline ECG for proband showing a saddle-shaped ST elevation in leads V1–V3.

A 7-month-old male infant was referred to our pediatric intensive care unit (PICU) in June 2017. He had a history of recurrent episodes of agitation and paleness accompanied by VT showed in ECG (Figure 1A). Blood tests showed a mild increase in cardiac troponin I (0.08 ng/ml) and B-type natriuretic peptide (BNP, 451 pg/ml). Electrolyte levels, blood glucose, liver, and kidney functions were all normal. Arterial blood gas analysis showed acidosis (pH: 7.253), while oxygen partial pressure, carbon dioxide partial pressure, and bicarbonate were normal. Echocardiography showed normal heart structure and function. Due to his incessant VT and invalid electric cardioversion, the boy had been treated with a variety of antiarrhythmic drugs including lidocaine, amiodarone, propafenone, metoprolol, and sotalol during the 2-month hospitalization period and was finally administrated successfully with propranolol at 3 mg/kg/day. His baseline ECG demonstrated ST elevation in V1–V3 at rest (Figure 1B), suggesting an atypical Brugada pattern. During more than 1-year follow-up, no more ventricular tachycardia reoccurred in this boy. Unfortunately, the boy had SCD half a year after stopping the administration of propranolol. His parents both had normal ECGs and echocardiography, and without family history of syncope or SCD.

### Whole-Exome Sequencing

Genomic DNA was extracted from peripheral blood following standard procedures. The study was performed according to the ethics committee guidelines of the Children's Hospital of Nanjing Medical University. Written informed consent was obtained from all patients and their parents. WES was performed on an Illumina HiSeq 2000 (Bio-Rad, Hercules, CA, USA) using 2 × 100-bp paired-end reads. Variants with allele frequencies higher than 1% were filtered out. The minor allele frequency (MAF) was annotated using databases dbSNP, 1,000 Genomes MAF (Chinese), ExAC, Genome Aggregation Database (gnomAD), and an in-house MAF database. The candidate variants were validated by Sanger sequencing and the pathogenicity of variants was annotated according to the American College of Medical Genetics and Genomics (ACMG) standards and guidelines.

### Direct Sequencing of the *SCN5A* Gene

To amplify exons of *SCN5A* genes, five primer pairs were designed (Supplementary Table 1). The PCR mixtures contained 1.5 μl of primers, 2.0 μl of DNA, 12.5 μl of 2 × Taq Master Mix (Vazyme Biotech Co., Ltd., Nanjing, China), and 9 μl of ddH_2_O in a total volume of 25 μl. Cycling conditions included a pre-denaturation step at 94°C for 5 min, followed by 34 cycles at 94°C for 30 s, 59°C for 30 s, and 72°C for 30 s, and a final extension at 72°C for 5 min. The PCR products were first purified and then sequenced using the BigDye Terminator v3.1 Cycle Sequencing Kit (Applied Biosystems, Foster City, CA, USA). In addition, 50 healthy, unrelated controls from the Chinese population were screened by Sanger sequencing to exclude novel variants as non-disease-associated variations. An *SCN5A* gene variant (GenBank accession number NM_000335) was used as a reference sequence.

### Minigene Plasmid Construction and Site-Directed Mutagenesis

To create hybrid minigene constructs, we used the pSPL3 minigene reporter vector to analyze the resultant mRNA transcripts. To perform the minigene assay, we generated fragments containing exon 14, where the variant was located, and 150–200 bp of flanking intronic regions with XhoI/BamHI restriction sites; these were amplified by PCR from the patient's genomic DNA. The forward primer contained a XhoI site: 5′accagaattctggagctcgagTGAGTGTCCCCAGGTCTAT, and the reverse primer contained a BamHI site: 5′tcaccagatatctgtggatccAGAAGCCGACCCTGAGATTC. The pSPL3 vector was digested by restriction enzymes XhoI/BamHI and then ligated with the purified PCR products to construct the wild-type (E14-WT) and mutant minigene (c.2262+3A>T) vectors using the ClonExpress II One Step Cloning Kit (Vazyme Biotech Co., Ltd.). All constructs were confirmed by bidirectional sequencing.

### Minigene Splicing Assay in HEK293, HeLa, and A549 Cells

HEK293, HeLa, and A549 cells were cultured in 12-well plates with 1 ml of DMEM in each well at 37°C in 5% CO_2_. When the confluence was 80–90%, cells were transfected with 1 μg of purified pSPL3, E14-WT, and c.2262+3A>T plasmids using Lipofectamine 2000 Transfection Reagent (Thermo Fisher Scientific, Waltham, MA, USA). After 24 h, the cells' total RNA was extracted using TRIzol (Thermo Fisher Scientific). The first cDNA strand was reverse transcribed using the HiScript III RT SuperMix for qPCR (Vazyme Biotech Co., Ltd.). The resulting cDNA was used as a template to amplify exon 3 with the SD6 forward primer (5′-TCTGAGTCACCTGGACAACC-3′) and SA2 reverse primers (5′- ATCTCAGTGGTATTTGTGAGC-3′). RT-PCR amplification of aberrant splice transcripts, agarose gel separation, and subsequent direct Sanger sequencing were performed.

### Construction of cDNAs Encoding *SCN5A* Mutants

The wild-type full-length human *SCN5A* cDNA (GenBank NM_000335) including 79 bp of retained mutant *SCN5A* cDNA was purchased and cloned into pcDNA3.1-3xFlag vectors using the ClonExpress Entry One Step Cloning Kit (Vazyme Biotech Co., Ltd.). Mutagenesis was confirmed by bidirectional sequencing.

### Western Blot Analysis

HEK293 cells transfected with wild-type or 79-bp-retaining mutant *SCN5A* cDNA-pcDNA3.1-3xFlag vectors were rapidly washed with ice-cold PBS, and whole-cell lysates were generated using lysis buffer containing protease inhibitors. The protein concentration was determined using the Micro BCA Protein Assay Kit (Thermo Fisher Scientific) with BSA as the standard. Then, 20 μg of total protein was separated by 10% SDS–PAGE and transferred onto a PVDF membrane. The membranes were blocked with TBS-T (0.1% Tween 20 in TBS) containing 5% non-fat milk for 1 h at room temperature and then incubated with primary anti-Flag (1:3,000; Sigma-Aldrich, St. Louis, MO, USA) and GAPDH (1:3,000; ProtTech Inc., Phoenixville, PA, USA) antibodies overnight at 4°C, followed by incubation with HRP-labeled secondary antibodies at room temperature for 1 h. Proteins were visualized by chemiluminescent detection.

## Results

**Figure 2:**
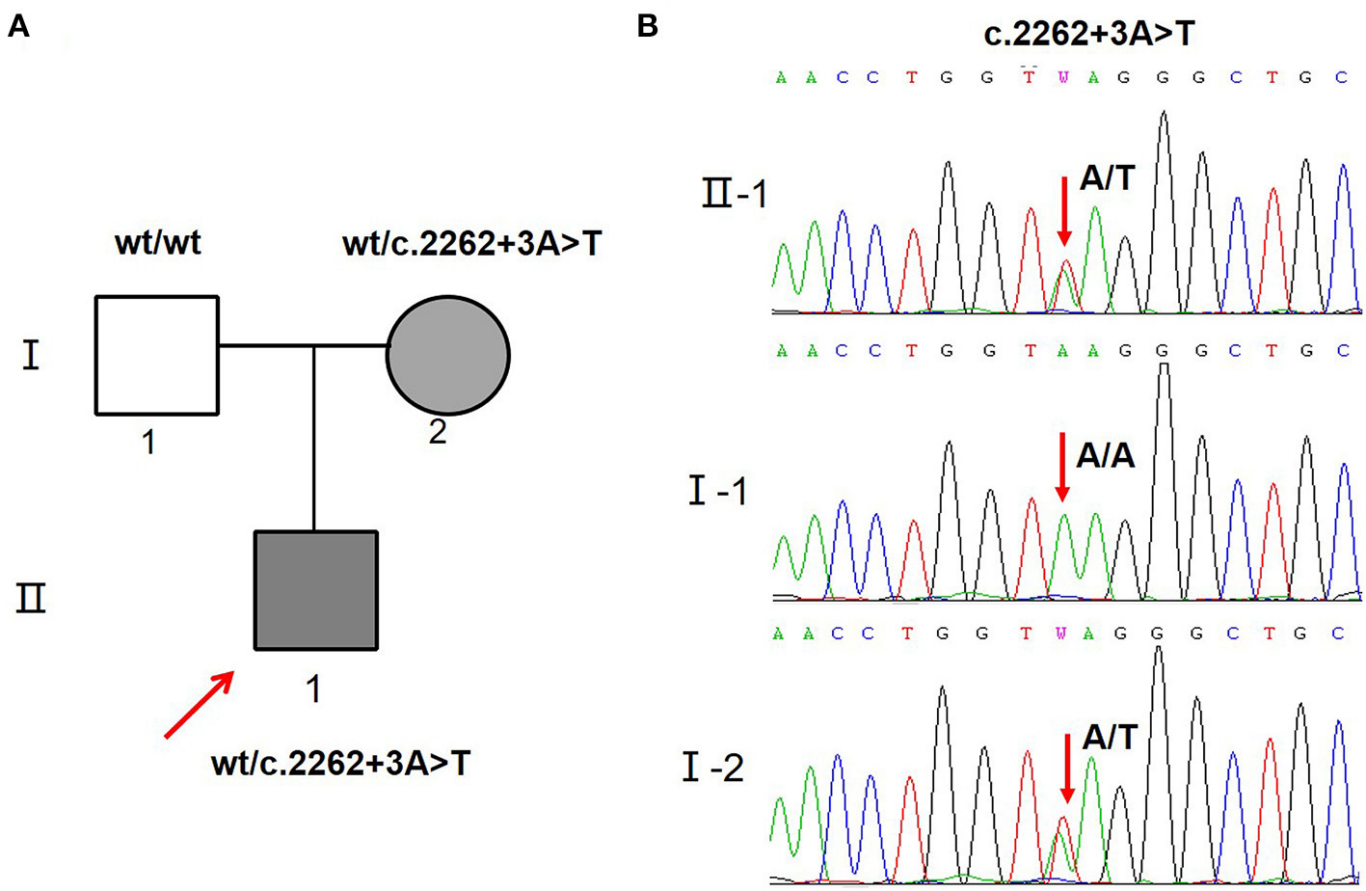
SCN5A gene variants identified in the proband and his family. **(A)** Schematic presentation of the familial pedigrees. Males and females are designated by squares and circles, respectively. The shapes filled with shadow indicate affected individuals with SCN5A mutation, arrow indicates proband. **(B)** Direct sequencing showing the novel variant c.2262+3A>T of the SCN5A gene.

A novel variant c.2262+3A>T located +3 bp from the splice donor site in intron 14 (Figure 2B) was identified in the proband and his mother (Figure 2A). According to the ACMG criteria, the c.2262+3A>T variant was supposed to be classified as VUS (PM2 + PP3). Human Splicing Finder analysis showed this variant is an “activation of an intronic cryptic acceptor site” (Supplementary Figure 1). Furthermore, another four splicing prediction tools (MaxEntScan, SpliceAI, NNSplice, and BDGP) also predicted that variant c.2262+3A>T disturbs the splice donor site (Supplementary Tables 2, 3).

**Figure 3:**
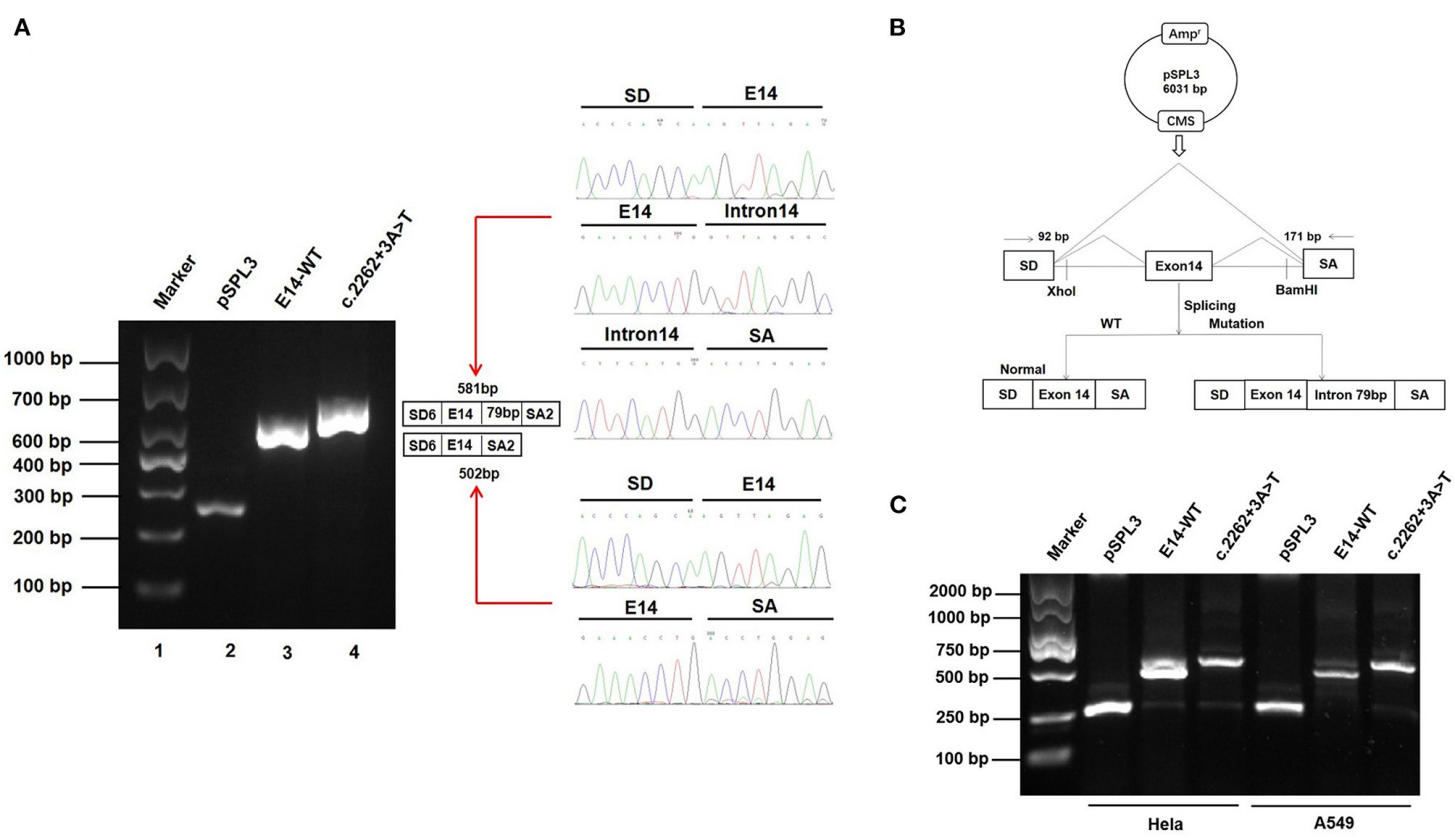
Effect of *SCN5A* gene c.2262+3A>T variant determined by minigene assays. **(A)** Gel electrophoresis of the RT-PCR products of minigene transcripts in HEK293 cells. Lane 1: marker; Lane 2: pSPL3 (263 bp); Lane 3: E14-WT (581 bp); Lane 4: c.2262 + 3A > T (502 bp). The two fragments were directly sequenced (right). **(B)** The transcripts produced by the hybrid minigene are shown schematically. **(C)** Gel electrophoresis of the RT-PCR products of minigene transcripts in HeLa and A549 cells.

**Figure 4:**
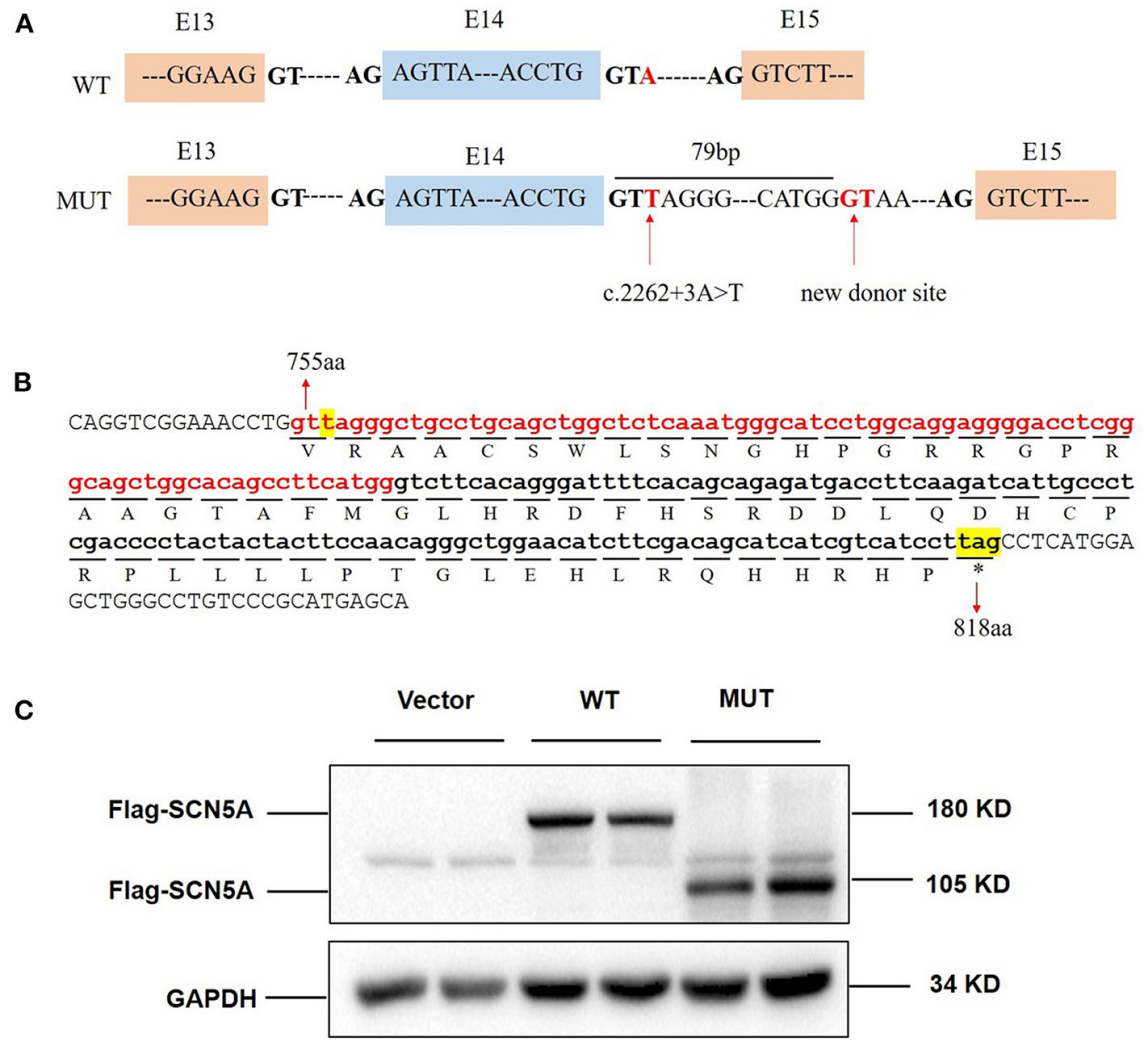
c.2262+3A>T variant caused protein truncation. **(A)** Exon 14 and adjacent structures in the *SCN5A* gene. The arrow shows the location of the splice site variant c.2262+3A>T and new donor site in intron 14. **(B)** Consequence of the c.2262+3A>T variant at the amino acid level. The retained 79 bp caused the addition of 63 amino acids followed by a premature stop codon at position 818. The protein translation is shown below the nucleotide sequence in the one-letter amino acid code. **(C)** SCN5A protein expression in whole HEK293 cells transfected with vector, wild-type, and mutant *SCN5A* plasmids.

Patient samples are not always available; minigene assays is an alternative strategy to evaluate the impact of splicing variants and represent powerful tools to investigate the regulation of splicing (17, 18). To further clarify the pathogenicity of the c.2262+3A>T variant, we performed RT-PCR splicing validation by construction of wild-type (E14-WT) and mutant minigene (c.2262+3A>T) vectors (Figure 3B) and transfected HEK293 cells with them. The E14-WT construct produced a full-length transcript of the expected size (502 bp), sequence, and structure (SD6, E14, and SA2). However, the c.2262+3A>T construct produced a larger transcript of 581 bp containing SD6, E14, SA, and the additional 79 bp. Subsequent sequence analysis revealed that the c.2262+3A>T mutant minigene caused aberrant splicing, resulting in the retention of the 79-bp sequence downstream of exon 14 (Figure 3A). To determine whether this transcriptional change also occurred in other cells, HeLa and A549 cells were also used. The c.2262+3A>T mutant minigenes produced larger transcripts containing SD6, E14, SA, and the additional 79 bp in these two cell lines, similar to HEK293 cells (Figure 3C). *In silico* analysis indicated that this mutant transcript would add 63 more amino acids to the original amino acids 755 and introduce a premature stop codon at amino acids 818 [p.(R818^*^)] (Figures 4A,B). Western blot analysis was performed to further confirm the production of a truncated protein by this variant. The wild-type *SCN5A* plasmid generated a 180 kDa protein, while the mutant 79-bp mutant *SCN5A* plasmid resulted in a low-molecular-weight band (~105 kDa) (Figure 4C).

## Discussion

The *SCN5A* gene encodes the alpha subunit of the Na_v_1.5 protein with four repeated transmembrane domains (DI–DIV) and is mainly expressed in the heart and highly abundant in working myocardium and conductive tissue (19). The Na_v_1.5 protein plays critical roles in cardiac electrical impulse conduction. Pathogenic variations in *SCN5A* gene result in a wide range of sodium channel dysfunctions.

In this study, we reported a young patient with recurrent incessant VT and SCD who carried a heterozygous splice variant in the *SCN5A* gene (c.2262+3A>T). He had atypical ST-segment elevation in the V1 to V3 leads on the ECG. *In silico* analysis suggested that c.2262+3A>T would disrupt the authentic splice donor site and activate the potential splice donor site of intron 14. Our minigene assay indicated that the c.2262+3A>T construct retained 79 bp at the 5′ end of intron 14 in the mature mRNA. This suggested that intron retention triggered by single-nucleotide alterations takes place in a variety of tissues. Normally, there are 2,015 amino acid residues in SCN5A. However, the transcript containing the 79-bp intron retention changed the structure and function of the protein, creating a premature stop codon at 818 amino acids [p.(R818^*^)].

Nonsense-mediated RNA decay (NMD) is an evolutionary conserved system of RNA surveillance that detects and degrades RNA transcripts containing nonsense mutations.

We predicted the c.2262+3A>T; p.(R818^*^) variant by the NMD prediction tool (https://nmdpredictions.shinyapps.io/shiny/), which showed that the p.(R818^*^) variant is subjected to degradation by NMD. However, in this study, *in vitro* overexpression analysis in HEK293 cells identified that the p.(R818^*^) variant produced a lower molecular-weight band (~105 kD) than that of the wild type (180 kD), suggesting that the transcripts derived from the p.(R818^*^) variant might escape from the NMD-control. Thus, we upgraded this variant to a likely pathogenic (PM2 + PP3 + PS3) one, and the c.2262+3A>T variant may be the pathogenic variant accounting for the VT of the proband. In general, variants cause the skipping of adjacent exons, while potential splicing sites are rarely activated. Hong et al. (20) produced the first report indicating the activation of a cryptic 5′ splice site resulting in the disruption of sodium channel activity in BrS; the proband was likely diagnosed with BrS. There were no events of VT or SCD in about 1-year treatment with propranolol, but unfortunately, he had an SCD after stopping the administration of propranolol for about half a year. Due to the young age of these five patients, we did not perform provocation test to detect Brugada-type ECG.

Studies of families with heritable arrhythmia with multiple mutation-positive individuals have shown low disease penetrance and clinical manifestations, including the types of symptoms, age of onset, and severity, which can be highly heterogeneous (21, 22). Age, sex, environmental factors, and comorbidities contribute to the phenotypic expression of arrhythmia (23). Furthermore, studies have found that genetic modifiers may be factors involved in interindividual phenotypic variability alongside the primary genetic defect. Although the mother of the patient carried the same *SCN5A* loss-of-function mutation, he had no history of syncope, arrhythmias, or SCD. The phenotype caused by loss of function type *SCN5A* variants is prevalent in adult male patients. Whether asymptomatic people who carried loss of function type *SCN5A* variants need drug treatment or ICD to prevent arrhythmias, is still controversial. Unfortunately, the proband suffered an SCD after stopping treatment. Therefore, pharmacological therapy like β-adrenergic, sometimes even ICD, may be necessary to protect patients who had history of malignant arrhythmia and carried SCN5A loss-of-function from SCD. In addition, his mother needs to be followed up for long time.

## Conclusion

In conclusion, we reported a novel splice variant c.2262+3A>T in the *SCN5A* gene in a young patient with recurrent incessant VT. This variant can impair the splicing donor site in intron 14, causing an insertion of 79 bp in the mRNA and producing a truncated protein (818 aa). This observation implies that a potentially pathogenic splicing variant is associated with incessant ventricular tachycardias.

## Data Availability Statement

The datasets presented in this article are not readily available due to ethical and privacy restrictions. Requests to access the datasets should be directed to the corresponding authors.

## Ethics Statement

The studies involving human participants were reviewed and approved by the study was performed according to the ethics committee of the Children's Hospital of Nanjing Medical University (Nanjing, China). Written informed consent was obtained from all the patients and their parents. Written informed consent to participate in this study was provided by the participants' legal guardian/next of kin.

## Author Contributions

SY and CW conceived and designed the study. JY and TX wrote the manuscript and performed the experiments. ZZ and CY collected the clinical samples and clinical data. JC and HZ wrote the clinical part of the manuscript. XC, BZ, and YQ performed the NGS analysis. SY and ZJ reviewed and edited the manuscript. All authors contributed to the article and approved the submitted version.

## Conflict of Interest

The authors declare that the research was conducted in the absence of any commercial or financial relationships that could be construed as a potential conflict of interest.

## Publisher's Note

All claims expressed in this article are solely those of the authors and do not necessarily represent those of their affiliated organizations, or those of the publisher, the editors and the reviewers. Any product that may be evaluated in this article, or claim that may be made by its manufacturer, is not guaranteed or endorsed by the publisher.
