# Supplementary material for: Case Report: A Novel Variant c.2262+3A>T of the SCN5A Gene Results in Intron Retention Associated With Incessant Ventricular Tachycardias
Source: Front Med (Lausanne). 2021 Aug 4;8:659119. doi: 10.3389/fmed.2021.659119 (PMC8371685; doi:10.3389/fmed.2021.659119)
Supplement: Supplementary file 1 [file Data_Sheet_1.docx]

**Table S1. Primers used for amplification and sequencing of the coding regions of the *SCN5A* gene.**

| **Variants** | **SCN5A region** | **Forward primer** | **Reverse primer** |
| --- | --- | --- | --- |
| c.2262+3A>T | Exon14 | ACTGTTGTCTTAAGCAGTTGTACG | TTCTGCACGCATTCTCCTCCACCTAC |

**Table S2. The BDGP and NNSpliceprediction of c.2262+3A>T variant.**

|  | BDGP | NNSplice |
| --- | --- | --- |
| WT | 1.00 | 0.90 |
| c.2262+3A>T | 0.82 | 0.61 |

**Table S3. The MaxEntScanprediction of c.2262+3A>T variant.**

|  | **Sequence** | **MAXENT** | **MDD** | **MM** | **WMM** |
| --- | --- | --- | --- | --- | --- |
| WT | CTGgtaagg | 9.68 | 13.48 | 8.54 | 10.10 |
| c.2262+3A>T | CTGgttagg | 3.35 | 6.88 | 3.57 | 5.15 |

**Table S4.** **Currently known 312 monogenic genes that are currently known to cause** **inherited arrhythmia.**

| AARS2 | ABCC6 | ABCC9 | ACAD8 | ACAD9 | ACADS | ACADVL | ACTA1 |
| --- | --- | --- | --- | --- | --- | --- | --- |
| ACTB | ACTC1 | ACTG1 | ACTN2 | AGK | AGL | AGXT | AKAP10 |
| AKAP9 | ALG1 | ALG10 | AMPD1 | ANK2 | ANKRD1 | ANKS6 | APOPT1 |
| ASAH1 | ATP5A1 | ATP5E | ATPAF2 | B3GALNT2 | B4GALT1 | B4GAT1 | BAG3 |
| BRAF | CACNA1C | CACNA1S | CACNA2D1 | CACNA2D4 | CACNB2 | CALM1 | CALM2 |
| CALM3 | CALR3 | CASQ2 | CAV3 | CBL | CBS | CDKN1C | CHKB |
| CHRM2 | COA5 | COA6 | COG7 | COQ2 | COQ4 | COQ9 | COX10 |
| COX14 | COX15 | COX20 | COX6B1 | CPT2 | CRYAB | CSRP3 | CTF1 |
| CTNNA3 | D2HGDH | DAG1 | DCAF8 | DCHS1 | DES | DMD | DNAJC19 |
| DOLK | DPM1 | DPM3 | DPP6 | DSC2 | DSG2 | DSP | DTNA |
| ELAC2 | EMD | EPG5 | EYA4 | FASTKD2 | FGF12 | FHL1 | FHL2 |
| FHOD3 | FKRP | FKTN | FLNC | FLT1 | FOXRED1 | FXN | GAA |
| GATA4 | GATA5 | GATA6 | GATAD1 | GBE1 | GFM1 | GJA1 | GJA5 |
| GLA | GLB1 | GMPPB | GNAI2 | GNPTAB | GNPTG | GPD1L | GPX4 |
| GSN | GTPBP3 | GUSB | GYG1 | GYS1 | H19 | HADH | HADHA |
| HAMP | HCN4 | HEXB | HEY2 | HFE | HFE2 | HRAS | IDH2 |
| IDS | IGHMBP2 | ILK | ISCU | ISL1 | ISPD | JPH2 | JUP |
| KAT6B | KCNA5 | KCND3 | KCNE1 | KCNE2 | KCNE3 | KCNE5 | KCNH2 |
| KCNJ2 | KCNJ5 | KCNJ8 | KCNK3 | KCNQ1 | KCNQ1OT1 | KCTD7 | KLF10 |
| KRAS | LAMA2 | LAMA4 | LAMP2 | LARGE1 | LDB3 | LIAS | LMNA |
| LPIN1 | LZTR1 | MAP2K1 | MAP2K2 | MCOLN1 | MGME1 | MIB1 | MLYCD |
| MRPL3 | MRPL44 | MRPS16 | MRPS22 | MTO1 | MYBPC3 | MYF6 | MYH6 |
| MYH7 | MYH7B | MYL2 | MYL3 | MYLK2 | MYO6 | MYOM1 | MYOT |
| MYOZ1 | MYOZ2 | MYPN | NAA10 | NAGA | NDUFA1 | NDUFA11 | NDUFAF1 |
| NDUFAF2 | NDUFAF3 | NDUFAF4 | NDUFAF5 | NDUFB11 | NDUFB3 | NDUFB9 | NDUFS1 |
| NDUFS2 | NDUFS3 | NDUFS4 | NDUFS6 | NDUFV1 | NDUFV2 | NEBL | NEXN |
| NF1 | NKX2-5 | NOS1AP | NOTCH3 | NPPA | NRAS | NSD1 | NSDHL |
| NUBPL | NUP155 | OBSCN | PDLIM3 | PDSS1 | PDSS2 | PET100 | PGM1 |
| PIGM | PITX2 | PKP2 | PLN | PMM2 | PNPLA2 | POMGNT1 | POMGNT2 |
| POMK | POMT1 | POMT2 | PPOX | PRDM16 | PRKAG2 | PRRX1 | PSEN1 |
| PSEN2 | PTPN11 | RAF1 | RANGRF | RBCK1 | RBM20 | RIT1 | RYR1 |
| RYR2 | SCN10A | SCN1B | SCN2B | SCN3B | SCN4A | SCN4B | SCN5A |
| SCO2 | SDHA | SDHAF3 | SGCD | SGCG | SHOC2 | SLC22A5 | SLC25A20 |
| SLC25A3 | SLC25A4 | SLC6A4 | SLMAP | SNTA1 | SOS1 | SOS2 | SPEG |
| SPRED1 | SRI | SYNE1 | SYNE2 | SYNM | TACO1 | TAZ | TBX5 |
| TCAP | TCF21 | TGFB3 | TMEM43 | TMEM5 | TMEM70 | TMPO | TNNC1 |
| TNNI3 | TNNI3K | TNNT2 | TNNT3 | TP63 | TPM1 | TPM2 | TRDN |
| TRIM37 | TRIM63 | TRMU | TRPA1 | TRPM4 | TSC1 | TSFM | TTN |
| TTR | TXNRD2 | VCL | VCP | WFS1 | XK | XPNPEP3 | ZFHX3 |

HSF

**
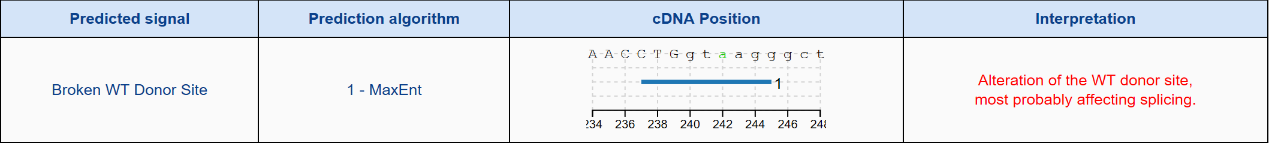
**

SpliceAI

**
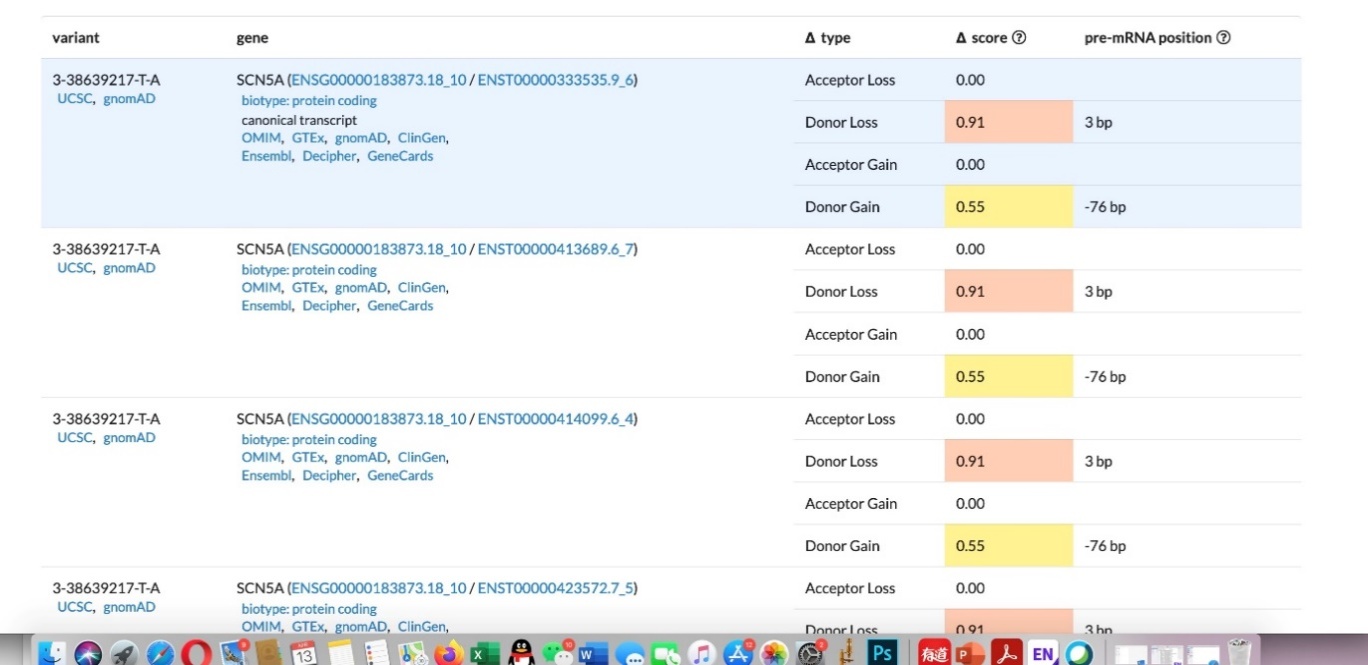
**

**Figure S1. HSF and SpliceAI prediction of c.2262+3A>T variant.**
